# Supplementary material for: Levetiracetam modulates brain metabolic networks and transcriptomic signatures in the 5XFAD mouse model of Alzheimer’s disease
Source: Front Neurosci. 2024 Jan 24;17:1336026. doi: 10.3389/fnins.2023.1336026 (PMC10847229; doi:10.3389/fnins.2023.1336026)
Supplement: Supplementary file 1 [file Data_Sheet_1.PDF]

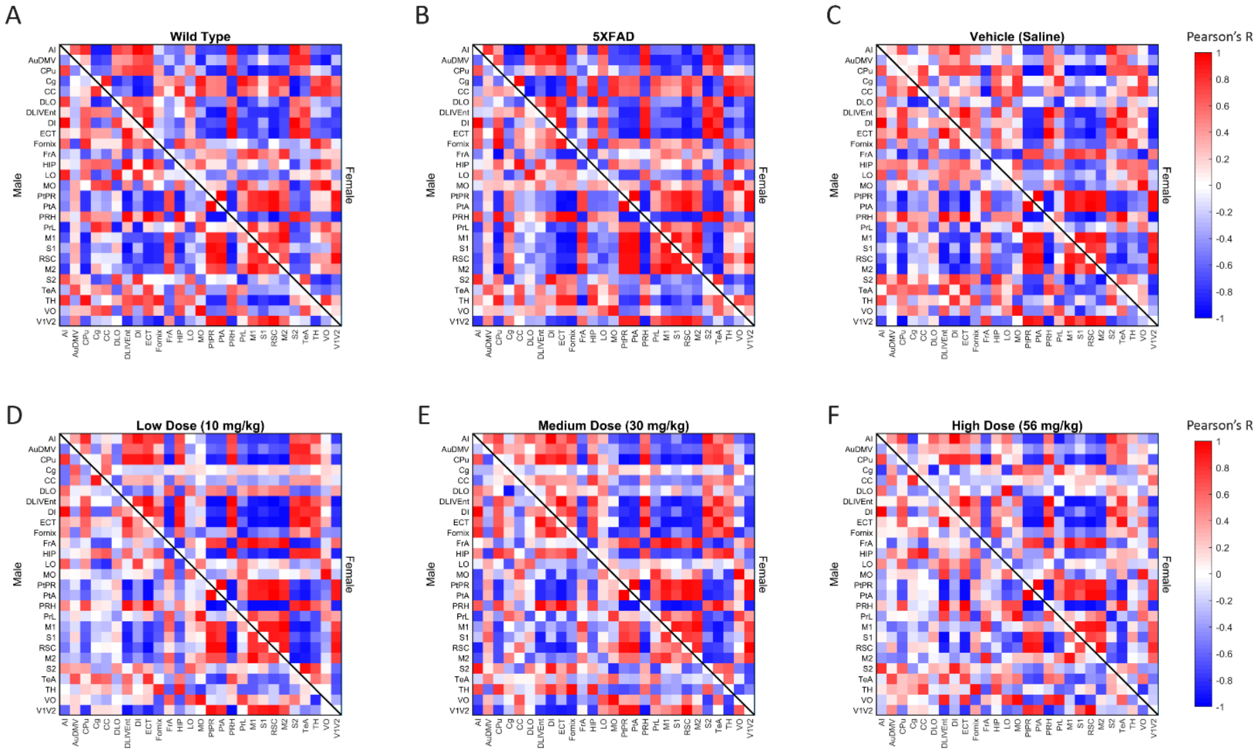

Supplementary Figure 1: Unthresholded metabolic covariance matrices sorted alphabetically by region.

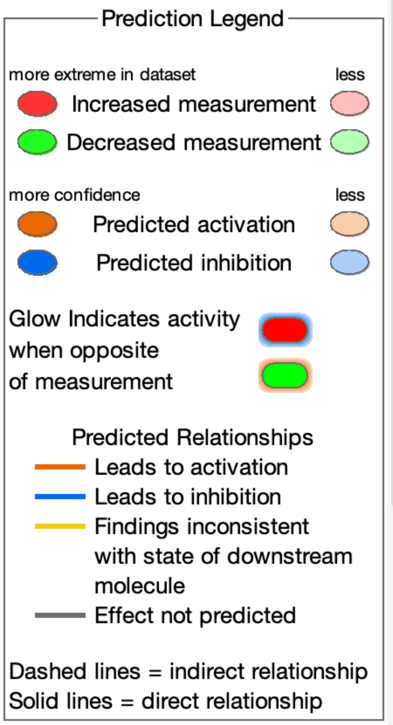

Supplementary Figure 2: Pathway prediction legend for GO term visualization (Figure 7B-D).

Index	Region of Interest (ROI) Name	ROI Label
1	Agranular Insular Cortex (Doral-Ventral)	AI
2	Auditory Cortex (Dorsal, Medial, Ventral)	AuDMV
3	Caudate Putamen (Dorsal Striatum)	CPu
4	Cingulate Cortex	Cg
5	Corpus Callosum	CC
6	Dorosolateral Orbital Cortex	DLO
7	Dorsintermed Entorhinal Cortex	DLIVEnt
8	Dysgranular Insular Cortex	DI
9	Entorhinal Cortex	ECT
10	Fornix	Fornix
11	Frontal Association Cortex	FrA
12	Hippocampus (CA1-CA3)	HIP
13	Lateral Orbital Cortex	LO
14	Medial Orbital Cortex	MO
15	Parietal Corext (Post-Rostral)	PtPR
16	Perietal Association Cortex (Lateral-Medial)	PtA
17	Perirhinal Cortex	PRH
18	Prelimbic Cortex	PrL
19	Primary Motor Cortex	M1
20	Primary Somatosenory Cortex	S1
21	Retrosplenial Dysgranular Cortex	RSC
22	Secondary Motor Cortex	M2
23	Secondary Somatosenory Cortex	S2
24	Temporal Association Cortex	TeA
25	Thalmus	TH
26	Ventral Orbital Cortex	VO
27	Visual Cortex (Primary and Secondary)	V1V2

Supplementary Table 1: Full names of network nodes (region of interest) as defined in the Paxinos and Franklin Atlas. Left and right regions were averaged to yield a network of 27 regions normalized by the cerebellum..
